# Successful Rescue of Massive Hemoptysis Caused by Vascular‐Bronchial Fistula

**DOI:** 10.1111/crj.70064

**Published:** 2025-02-27

**Authors:** Xiangwu Zhang, Rongxian Zhou, Guangqiang Zhao, Wanling Chen, Yunchao Huang, Lianhua Ye

**Affiliations:** ^1^ Department of Thoracic Surgery The Third Affiliated Hospital of Kunming Medical University (Yunnan Cancer Hospital) Kunming China; ^2^ School of Rehabilitation Medicine Yunnan Institute of Economics and Management Kunming China

**Keywords:** hemoptysis, rescue, vascular‐bronchial fistula

## Abstract

The case reported in this paper is a vascular‐bronchial fistula associated with fatal massive hemoptysis. The patient was rescued successfully by experts from a multidisciplinary team. The bronchoscopy physician cleared the accumulated blood in the airway timely and maintained the airway patency, and the anesthesiologist established artificial ventilation quickly; eventually, the thoracic surgeon performed an emergency thoracotomy to control the bleeding. It reflected the significance of multidisciplinary collaborative treatment.

## Graphical abstract

The case reported in this paper is a vascular‐bronchial fistula associated with fatal massive hemoptysis. The patient was successfully rescued by experts from a multidisciplinary collaborative team. The bronchoscopy physician timely cleared the accumulated blood in the airway and maintained the airway patency, and the anesthesiologist quickly established artificial ventilation; eventually, the thoracic surgeon urgently performed a thoracotomy to control the bleeding. This reflects the significance of multidisciplinary collaborative treatment.

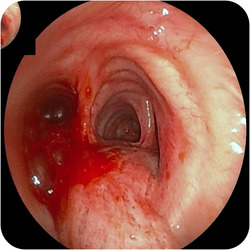

## 1 Introduction

Vascular‐bronchial fistula associated with massive hemoptysis is an extremely critical and life‐threatening clinical emergency that is fatal if untreated. Therefore, timely diagnosis and accurate rescue are particularly important. There are few successful rescued cases of hemoptysis caused by vascular‐bronchial fistula. This paper reports the successful case of fatal massive hemoptysis. It aims to provide a reference for the rescue of critically massive hemoptysis for the multidisciplinary collaborative team.

## 2 Case Report

**FIGURE 1:**
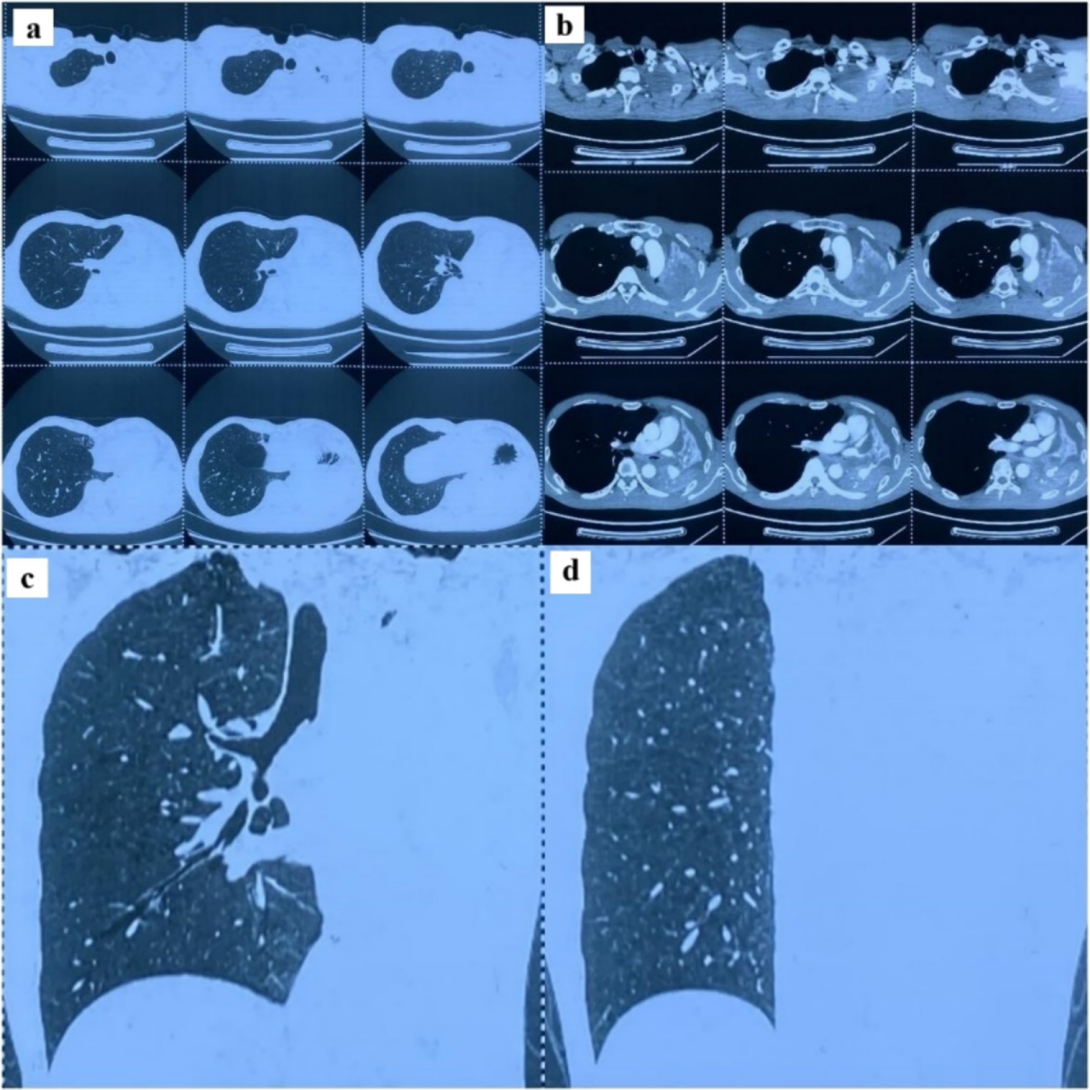
CT showed left total atelectasis and left main bronchus obstruction. (a) Horizontal pulmonary window. (b) Horizontal septum longitudinale window. (c,d) Coronal position.

**FIGURE 2:**
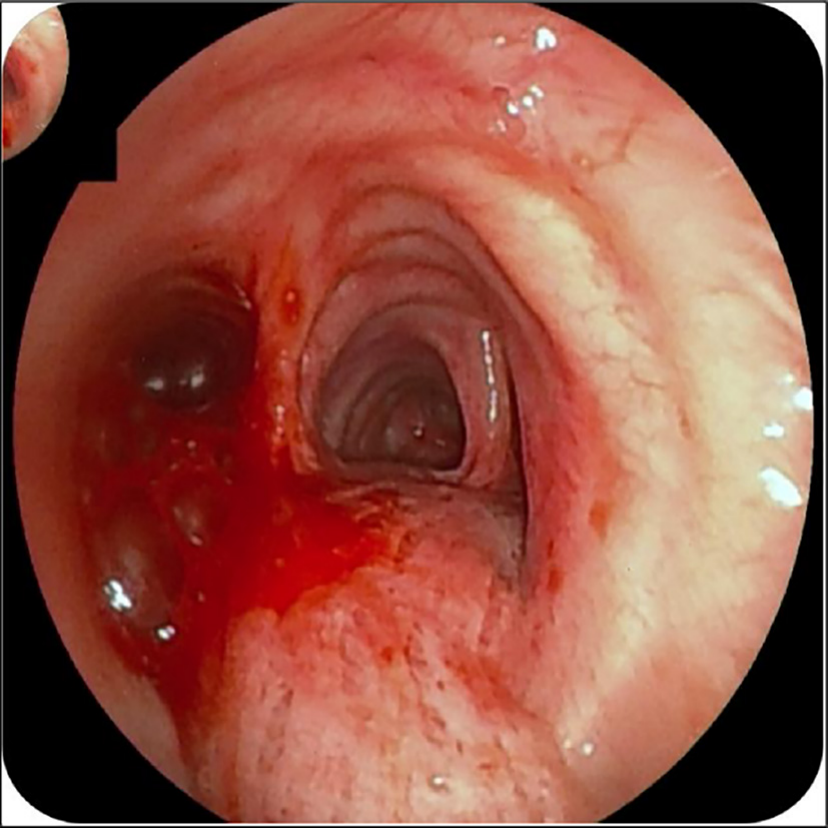
Bronchoscopy showed a large amount of hemorrhage from the left main bronchus.

**FIGURE 3:**
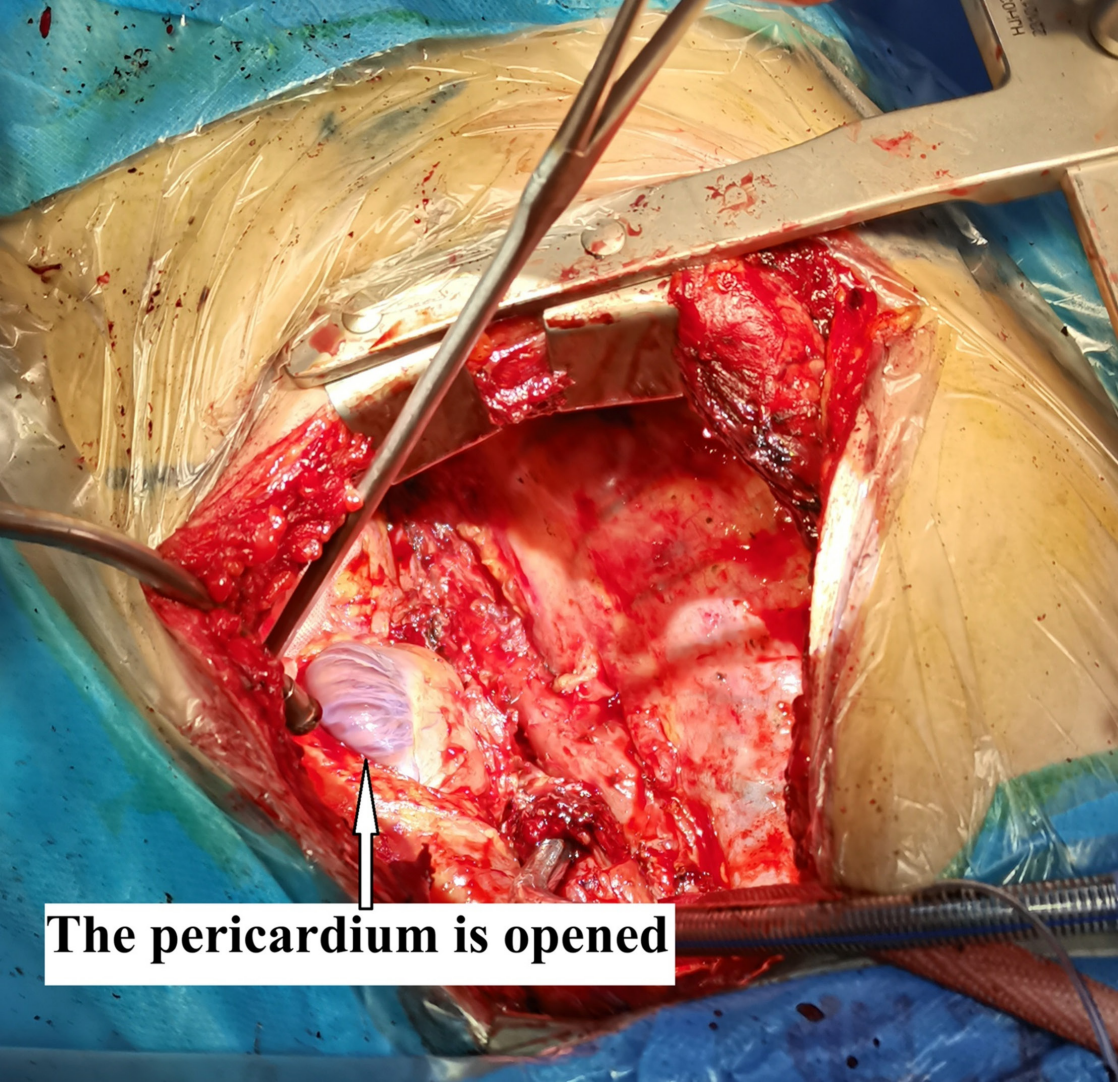
The pericardium is opened.

**FIGURE 4:**
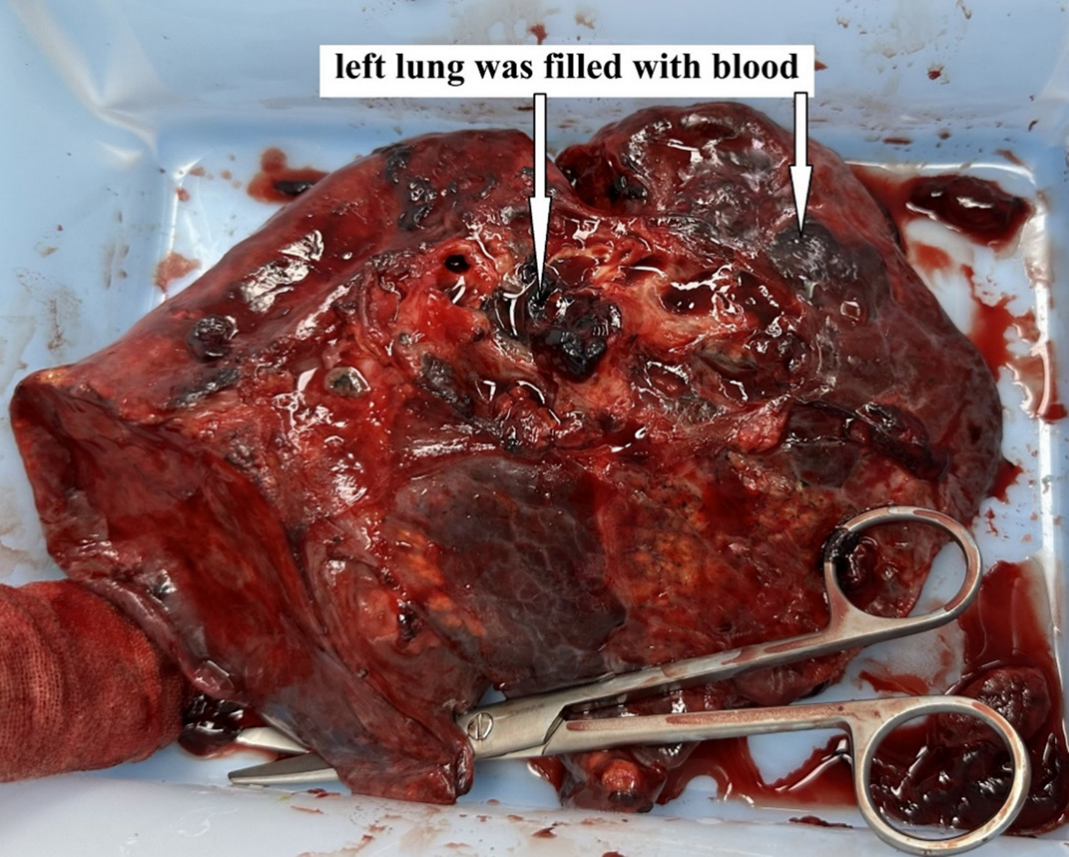
The left lung was filled with blood.

**FIGURE 5:**
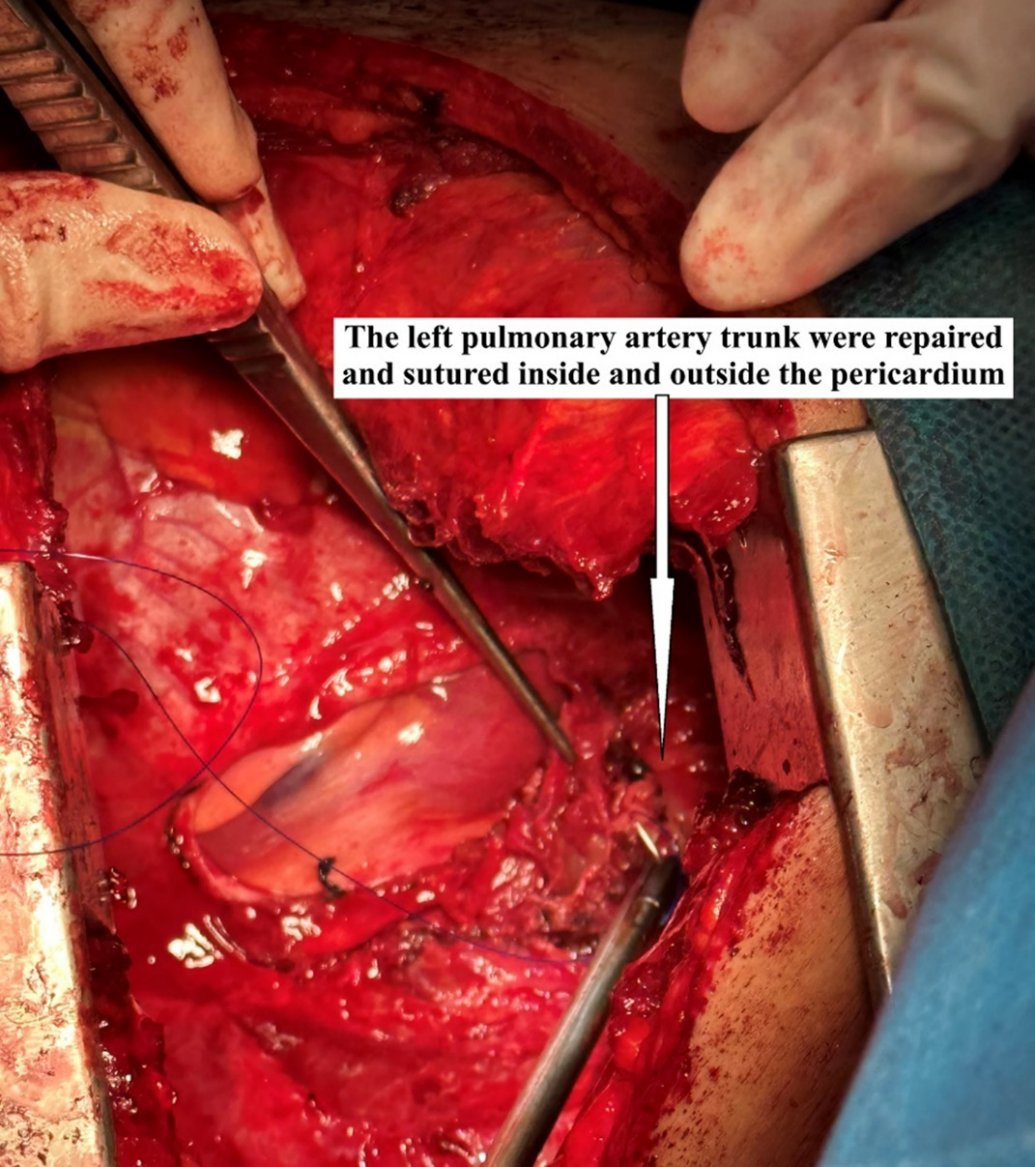
The wall of the pulmonary artery trunk and the left pulmonary artery trunk were repaired and sutured inside and outside the pericardium.

**FIGURE 6:**
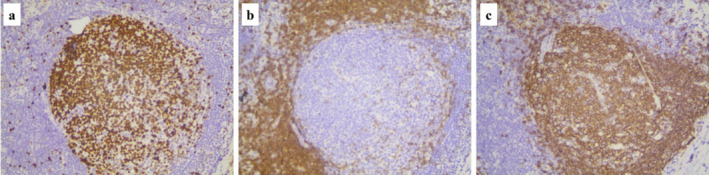
The pathology, immunohistochemical results. (a) The diseased tissue was positive for Ki67. (b) The diseased tissue was positive for CD3(T cell). (c) The diseased tissue was positive for CD20(B cell).

A 40‐year‐old man had hemoptysis and progressive dyspnea for 1 month, which aggravated with massive hemoptysis for 3 days. The patient history is as follows: He was a sanitation worker with no history of smoking, tuberculosis exposure, heart and aortic surgery, pseudoaneurysm, bronchiectasis, and family tumor‐related diseases. He was in poor general condition, and his blood pressure (BP) was 101/60 mmHg, oxygen saturation (SPO_2_) was 70%, and heart rate was 127 bpm. Computed tomography (CT) showed left total atelectasis and left main bronchus obstruction (Figure 1). First, we tried to use a bronchoscope (FUJIFILM EB‐580T, working channel diameter is 2.8 mm) to clear airway congestion and stop bleeding. Bronchoscopy showed hemorrhage from the left main bronchus (Figure 2), but we failed to compress it with a balloon due to the heavy bleeding. So his BP and SPO_2_ decreased. Next, our multidisciplinary team took an urgent rescue. The anesthesiologist rapidly established a double‐lumen endotracheal tube to maintain ventilation of the right main bronchus because of the high endobronchial pressure, and we failed to block through a double‐lumen endotracheal tube and compress with a balloon. The situation worsened, and lots of blood flowed into the right main bronchus. Besides, bloody secretions in the airway were aspirated via the bronchoscope to prevent the right lung from being submerged. At this point, we had to control the bleeding by an emergency left thoracotomy, which revealed that the left lung was filled with blood in the airway resulting in a solid lung. The bleeding was slightly reduced when the left lung portal with blocking forceps. After opening the pericardium (Figure 3), the left superior pulmonary vein, pulmonary trunk, and left main pulmonary artery in the pericardium adhered to the left main bronchus. Then, the left main bronchus was incised. Loosening the blocking forceps, a large amount of blood gushed out; meanwhile, it was observed that the left main pulmonary artery communicated with the left main bronchus to form a left main pulmonary artery‐left main bronchus fistula. The left main pulmonary artery trunk and the left wall of the pulmonary artery trunk were intermittently clamped to remove the left whole lung quickly (Figure 4). The wall of the pulmonary artery trunk and the left pulmonary artery trunk were repaired and sutured inside and outside the pericardium (Figure 5). Through the efficient, rapid, and accurate rescue, the BP and SPO_2_ of the patient increased and gradually stabilized, although the loss of blood was nearly 8000 mL. Pathology suggested lymphoid tissue hyperplasia. Immunohistochemical results showed CK(−), Vim(+), Ki67(+), CD3(T cell+), CD20(B cell+), CD5(T cell+), CD79α(B cell+), PAX5(follicle+), BcL2(−), BcL6(germinal center+), CyclinD1(−), MUM1(focus+), CD43(+), CD34(vas+), CD38(focus+), CD138(focus+), MPO(−), CD21(+), CD23(FDC net+). Combined with hematoxylin–eosin (HE) and immunohistochemistry, it was consistent with reactive hyperplasia of lymph nodes (Figure 6). After the operation, we consistently removed the hematocele in each bronchus of the right lung via the bronchoscope. The tracheal intubation was removed on the third day, and he recovered and was discharged successfully on the eighth day. And he was followed up by telephone in the half a month, the first month, and the third month after the operation. There was no hemoptysis and he returned to normal work and life.

## 3 Discussion

Hemoptysis is a common clinical symptom, often caused by bleeding from the bronchial arteries or other blood vessels around the tracheobronchial tract. In most cases, hemostasis can be achieved by local application of vasoconstrictors, balloon compression, or bronchial artery embolization. This patient's left whole lung had consolidated due to repeated massive hemoptysis. The sudden massive hemorrhage in the airway could not be compressed by the balloon and seriously threatened the patient's life. Multidisciplinary collaborative emergency treatment was performed, and it was demonstrated that the left main pulmonary artery communicated with the left main bronchus to form a vascular‐bronchial fistula during the operation.

Vascular‐bronchial fistula, which results from abnormal communication between vessels and the bronchial tree, is rare and complicated, usually manifested as massive hemoptysis with a high risk of asphyxia death [1]. It is mostly related to multiple interventions, including thoracic aortic aneurysm surgery, lung transplantation, anastomotic complications after sleeve pneumonectomy, airway stent placement with erosion, and radiotherapy. The pathogenesis may be related to infection, thrombosis, vascular reconstruction, and other factors [2, 3]. There was no specific cause in this case, and it was a fatal massive hemoptysis of unexplained origin. Finally, the operation was confirmed to be pulmonary‐bronchial fistula, and the pathological diagnosis was reactive lymphoid hyperplasia. The pulmonary lymphatic system consists of bronchial‐associated lymphoid tissue (BALT) and pulmonary lymphatic vessels. Reactive lymphoid hyperplasia is one of the primary pulmonary lymphadenopathy, caused by BALT [4]. BALT does not exist at birth, develops in infancy and early childhood, and does not exist in normal healthy adults. BALT can be induced by adult antigen stimulation, such as infection, smoking, dust, chronic inflammation, collagen vascular disease, and AIDS. The patient was engaged in sanitation work and had a long history of dust exposure, which induced the formation of BALT, leading to reactive lymphoid hyperplasia, invasion and destruction of the hilar structure, leading to pulmonary artery bronchial fistula, and ultimately leading to massive hemoptysis, which may be the cause of the case.

Vascular‐bronchial fistula associated with massive hemoptysis is an extremely critical and life‐threatening clinical emergency that is fatal if untreated. Timely diagnosis and accurate rescue are greatly crucial. Digital subtraction angiography (DSA) is the gold standard for the identification of vascular‐bronchial fistula, and computed tomography angiography (CTA) allows direct visualization of the fistula [5]. However, due to the obvious symptoms of hemoptysis and the critical condition, visualization is rarely possible via CTA in this type of patient in clinical practice [6]. Therefore, the patient has a history of cardiac or aortic surgery, pseudoaneurysm, and massive hemoptysis suggesting urgent endobronchial or surgical treatment [7].

Blood clots in the airway often lead to death from asphyxia, and the airway must be managed immediately to maintain airway patency. First, the patient should be immediately placed in the affected lateral position to prevent ventilation of the unaffected lung from being affected. Next, the bronchoscopy physician cleared the accumulated blood timely, maintained the airway patency, and locally used vasoconstrictors and balloons to block the compression. It should be noted that bronchoscopy may stimulate a severe cough and lead to an increase in hemoptysis, so bronchoscopy is recommended to be performed in an operating room equipped with thoracotomy conditions and tracheal intubation should be prepared in advance. If it can not be compressed with the balloon, the anesthesiologist should establish artificial ventilation quickly, and it is recommended to prepare extended tracheal intubation so that the tracheal tube can be inserted into the main bronchus of the healthy side in case of emergency to ensure ventilation. During tracheal intubation, a large amount of hemoptysis may affect the visual field, resulting in unsuccessful intubation. Electronic bronchoscopy can be used to guide rapid tracheal intubation. If the symptoms of hemoptysis are not obvious, or the amount of bleeding is not a threat to the patient's life, the fistula can be blocked by vascular intervention under DSA. Emergency thoracic intervention is the only option if compression and blocking can not be performed due to a large fistula. Given the extremely high mortality rate of this emergency procedure, timely diagnosis and accurate rescue are crucial. This case had a large amount of fresh blood that poured into the trachea and right main bronchus from the left main bronchus. The situation was very dangerous. The thoracic surgeon urgently opened the chest to control the bleeding, and the patient turned to safety.

As there are many predictable factors in the successful rescue cases reported in the past, the successful rescue experience is not very informative for the rescue of fatal massive hemoptysis of unexplained origin. The case reported in this paper is a fatal massive hemoptysis of unexplained origin. However, the patient was rescued successfully by a multidisciplinary collaborative team, which fully reflects the significance of multidisciplinary team treatment, the criticality of the timely establishment of an airway, and the maintenance of airway patency as much as possible to avoid severe hypoxemia and asphyxia in the case of airway hemorrhage to gain time for surgical operation. It provides a reference for the rescue of critically massive hemoptysis for the multidisciplinary collaborative team.

## Author Contributions

Organize Emergency Rescue: Lianhua Ye, Yunchao Huang, Guangqiang Zhao. Case Data Collection: Xiangwu Zhang, Rongxian Zhou. Manuscript writing: Xiangwu Zhang, Rongxian Zhou. Final approval of manuscript: Guangqiang Zhao, Wanling Chen, Lianhua Ye. All authors contributed to and approved the final version of the manuscript.

## Ethics Statement

This paper reports aims to provide a reference for the rescue of critically massive hemoptysis for the multidisciplinary collaborative team. The research was conducted in accordance with ethical guidelines, ensuring the protection of patients' rights and confidentiality. The patient signed preoperative informed consent for surgery. In addition, the patient consented to the use of his related data for academic publication after de‐identification.

## Consent

Signed authorization was obtained from the patient's legal representative giving consent to publish the information disclosed in this case report.

## Conflicts of Interest

The authors declare no conflicts of interest.

## Data Availability

The data that support the findings of this study are available from the corresponding author upon reasonable request.
